# Correction to: Volume 2 Issue 1 of *PNAS Nexus*

**DOI:** 10.1093/pnasnexus/pgad016

**Published:** 2023-01-27

When Volume 2, Issue 1, January 2023 of *PNAS Nexus* was opened, it was incorrectly labeled March 2023. The month has been corrected, and citation details of the following list of articles were affected by this error:

Cystic fibrosis rabbits develop spontaneous hepatobiliary lesions and CF-associated liver disease (CFLD)-like phenotypes. https://doi.org/10.1093/pnasnexus/pgac306

High-resolution cryo-EM structure of the junction region of the native cardiac thin filament in relaxed state. https://doi.org/10.1093/pnasnexus/pgac298

Topical SCD-153, a 4-methyl itaconate prodrug, for the treatment of alopecia areata. https://doi.org/10.1093/pnasnexus/pgac297

Development of an in-vitro high-throughput screening system to identify modulators of genitalia development. https://doi.org/10.1093/pnasnexus/pgac300

2,3,7,8-Tetrachlorodibenzo-p-dioxin induces multigenerational alterations in the expression of microRNA in the thymus through epigenetic modifications. https://doi.org/10.1093/pnasnexus/pgac290

Cell-based analysis reveals that sex-determining gene signals in Ostrinia are pivotally changed by male-killing *Wolbachia*. https://doi.org/10.1093/pnasnexus/pgac293

Information maximization explains state-dependent synaptic plasticity and memory reorganization during non-rapid eye movement sleep. https://doi.org/10.1093/pnasnexus/pgac286

Pharmacological potentiators of the calcium signaling cascade identified by high-throughput screening. https://doi.org/10.1093/pnasnexus/pgac288

Leaded aviation gasoline exposure risk and child blood lead levels. https://doi.org/10.1093/pnasnexus/pgac285

On the interplay of hierarchies, conflicts, and cooperation: An experimental approach. https://doi.org/10.1093/pnasnexus/pgac283

Senescent stroma induces nuclear deformations in cancer cells via the inhibition of RhoA/ROCK/myosin II-based cytoskeletal tension. https://doi.org/10.1093/pnasnexus/pgac270

On shape forming by contractile filaments in the surface of growing tissues. https://doi.org/10.1093/pnasnexus/pgac292

Customizable, reconfigurable, and anatomically coordinated large-area, high-density electromyography from drawn-on-skin electrode arrays. https://doi.org/10.1093/pnasnexus/pgac291

Watershed memory amplified the Oroville rain-on-snow flood of February 2017. https://doi.org/10.1093/pnasnexus/pgac295

A deep-learning model of prescient ideas demonstrates that they emerge from the periphery. https://doi.org/10.1093/pnasnexus/pgac275

Risky cascading transitions in international relationships. https://doi.org/10.1093/pnasnexus/pgac289

Moralized language predicts hate speech on social media. https://doi.org/10.1093/pnasnexus/pgac281

The citation details for each of the above articles have been updated to reflect the correct month of this issue. The Publisher regrets this error.

